# Development of a Double Tuned ^2^H/^31^P Whole‐Body Birdcage Transmit Coil for ^2^H and ^31^P MR Applications From Head to Toe at 7 T

**DOI:** 10.1002/nbm.5325

**Published:** 2025-01-31

**Authors:** Ayhan Gursan, Busra Kahraman‐Agir, Mark Gosselink, Dimitri Welting, Martijn Froeling, Hans Hoogduin, Evita C. Wiegers, Jeanine J. Prompers, Dennis W. J. Klomp

**Affiliations:** ^1^ Center for Image Sciences University Medical Center Utrecht Utrecht The Netherlands; ^2^ Departments of Human Biology and Imaging, NUTRIM Institute of Nutrition and Translational Research in Metabolism Maastricht University Medical Centre+ Maastricht The Netherlands

**Keywords:** 7 T, deuterium metabolic imaging, energy metabolism, glucose metabolism, liver

## Abstract

Deuterium (^2^H) and phosphorus (^31^P) magnetic resonance spectroscopy (MRS) are complementary methods for evaluating tissue metabolism noninvasively in vivo. Combined ^2^H and ^31^P MRS would therefore be of interest for various applications, from cancer to diabetes. Loop coils are commonly used in X‐nuclei studies in the human body for both transmit and receive. However, loop coils suffer from limited penetration depth and inhomogeneous B_1_^+^ field. The purpose of this work is to develop a double tuned ^2^H/^31^P whole‐body birdcage transmit coil for 7 T for ^2^H and ^31^P MRS imaging (MRSI) with homogeneous excitation over a large field‐of‐view. The performance of the ^2^H/^31^P birdcage coil was assessed on B_1_^+^ fields over a body‐sized phantom at ^2^H and ^31^P frequencies using an 8‐channel ^2^H/^31^P receive array. Using two elements of the ^2^H/^31^P receive array, natural abundance ^2^H and ^31^P 3D MRSI data at rest were acquired consecutively in the brain and lower leg muscles. Additionally, ^2^H and ^31^P 3D MRSI data were acquired from one volunteer 90 min after [6,6′‐^2^H_2_]‐glucose intake, using 8‐channel ^2^H/^31^P receive array around the abdomen. The B_1_^+^ variation of the whole‐body birdcage coil over the phantom was 12.1% for ^2^H and 19.2% for ^31^P. High‐quality ^2^H and ^31^P 3D MRSI data were acquired from the brain and the lower leg. Whole liver coverage was achieved in both ^2^H and ^31^P 3D MRSI data. The developed ^2^H/^31^P whole‐body birdcage transmit coil allows simultaneous 3D mapping of glucose and energy metabolism and membrane turnover throughout the human body.

## Graphical abstract

The development of a double tuned ^2^H/^31^P whole‐body birdcage transmit coil for 7 T allows the combined ^2^H and ^31^P MRSI acquisition, for simultaneous mapping of glucose energy metabolism and membrane turnover. The feasibility of obtaining ^2^H and ^31^P MRSI in the brain, liver and lower leg was demonstrated.

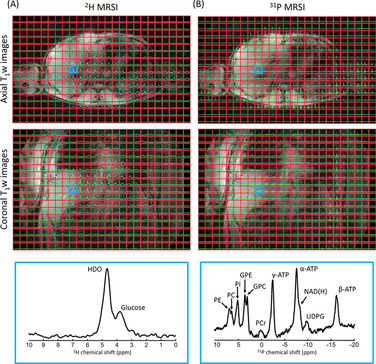

**Abbreviations**
AFI: actual flip angle imaging
EM: electromagnetic
FOV: field of view
RF: radiofrequency
SAR: specific absorption rate
PDE: phosphodiesters
PME: phosphomonoesters
pSAR: peak specific absorption rate
UDPG: uridine diphosphate glucose
VOI: volume of interest

## 1 Introduction

Phosphorus (^31^P) and deuterium (^2^H) magnetic resonance spectroscopy (MRS) are powerful tools to assess tissue metabolism in vivo in health and disease [1, 2, 3]. Both techniques are capable of detecting aberrant metabolism in tumor tissue: increased cell proliferation in tumors leads to an increased ratio of phosphomonoesters‐to‐phosphodiesters, detectable by ^31^P MRS [4, 5], whereas the Warburg effect results in an increased production of lactate in tumors, which dynamics can be quantified with ^2^H MRS after administration of deuterated glucose [1]. Therefore, the combined application of ^2^H and ^31^P MRS in humans yields complementary information and would be of interest at various locations in the human body, ranging from the brain to the body and extremities.

Double tuned coils have been developed for varying nuclei for MR applications in humans, specifically for use at ultra‐high field, to benefit from its increased sensitivity [6]. Double tuned coils had to be used for both RF transmit and receive for X‐nuclei, as there is no default X‐nuclei RF transmit capability in MRI scanners, such as there is for ^1^H in clinical scanners at 1.5 and 3 T. Furthermore, double tuned coils have mainly been built for specific applications in humans such as for the brain [7, 8, 9], abdomen [10], or skeletal muscle [7, 11]. These coils were optimized for the geometry and electrical load of the organ of interest and assessed in terms of safety to stay within local peak specific absorption rate (pSAR) limits. Hence, these coils cannot always be used freely in studying other organs. As a result, for almost every new X‐nuclei study, a new coil has to be built and assessed for safety, which can be costly and time inefficient. Moreover, although examples of birdcage coils for X‐nuclei exist [7, 12, 13, 14], because of ease in design (surface) loops are more often used in X‐nuclei studies [8, 11, 15, 16, 17]. Loop coils benefit from higher sensitivity, in the perimeter of the coil, compared with birdcage coils, but at the cost of an inhomogeneous B_1_^+^ field [18]. This B_1_^+^ inhomogeneity results in a reduction in effective flip angles at larger distances from the loop coil, and T_1_ saturation will be different with respect to the distance to the coil. Consequently, quantification of metabolites will become unreliable when T_1_ relaxation times vary between the metabolites [19, 20, 21]. Using long repetition times (TR), one could reduce T_1_ saturation, but this comes at the cost of long scan durations. Flip angle homogeneity can be achieved to an extent by using adiabatic pulses [22]. However, adiabatic RF pulses are much longer than conventional (block) pulses and therefore require the use of substantially longer TR to remain within SAR guidelines.

In recent studies, the feasibility of using a ^31^P whole‐body birdcage coil at ultra‐high field has been demonstrated [12, 23]. Compared with loop coils with adiabatic RF pulses, pSAR values of birdcage coils are lower; hence, fast 3D MRSI acquisitions become feasible with low flip angle and short TR acquisitions without compromising signal coverage of deeper tissues in the body [24]. In combination with a 16‐channel receiver array, the whole‐body birdcage coil provided full coverage of the largest organ in the body (liver) with good to excellent repeatability over the quantification of ^31^P metabolites over the entire liver [25]. Furthermore, the acquisition of pancreas ^31^P MRSI became feasible with this combination, which was challenging to acquire with loop coil because of limited penetration depth [26].

Due to spatial constrains within the MRI scanners, especially at ultra‐high field, accommodating two single tuned birdcage coils for ^2^H and ^31^P is impractical. In this study, we developed a double tuned ^2^H/^31^P whole‐body birdcage transmit coil for 7 T, for ^2^H and ^31^P MRS with homogeneous excitation over a large field‐of‐view and with the increased sensitivity offered by ultra‐high field. Here, we characterize the ^2^H and ^31^P B_1_^+^ fields of the whole‐body birdcage transmit coil and demonstrate the feasibility of combined ^2^H and ^31^P 3D MRSI measurements of the brain, liver and lower leg, using a ^2^H/^31^P receive array.

## 2 Methods

### 2.1 Hardware

**FIGURE 1:**
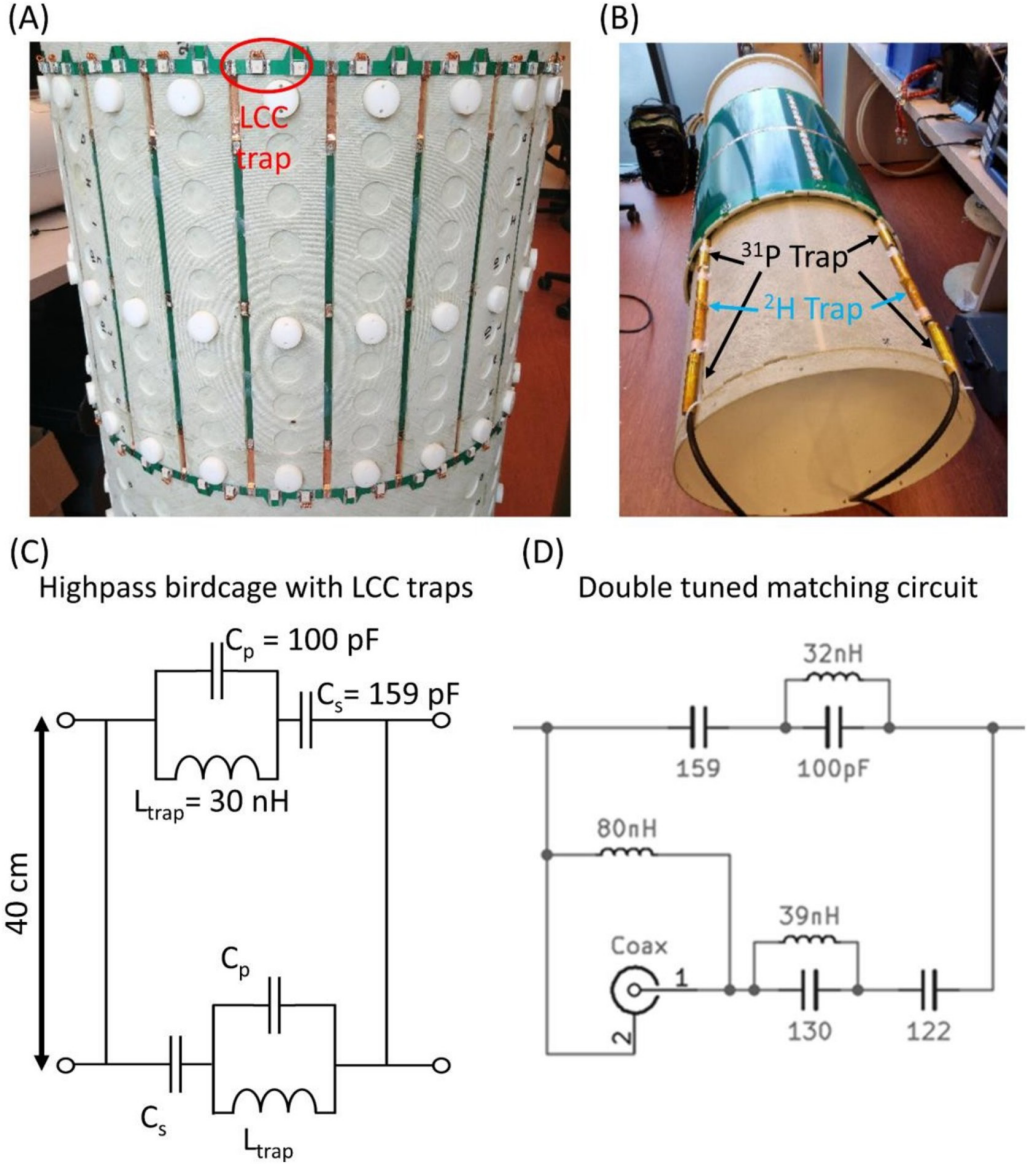
Developed 24‐rung double tuned ^2^H/^31^P whole‐body birdcage transmit coil without (A) and with (B) shield, before installation in the 7 T MRI system. Within the red circle, one of the LCC trap circuits is highlighted (A). Schematic of highpass double tuned birdcage coil design with LCC trap circuits between two of the rods (C) and matching circuit (D).

A shielded, 24‐rung, high‐pass double tuned ^2^H/^31^P whole‐body birdcage transmit coil [27] with a diameter of 60 cm and a length of 40 cm, embedded in the outside of the patient tube (Futura, Heerhugowaard, the Netherlands) of the MRI system, was constructed (Figure 1A,B). The coil rod and ring geometries were matched to a commercial 3 T MRI system and was tuned to 45.7 and 120.6 MHz for ^2^H and ^31^P, respectively, at 7 T, by using LCC traps on the end rings, between the rungs (Figure 1C). LCC traps consisted of C_s_ = 159 pF, C_p_ = 100 pF and a hand wound inductor L_p_ = 30 nH that sets the trap frequency between 45.7 and 120.6 MHz. The shield was composed of eight segmented PCBs, each 493.55 × 339.85 mm^2^. The distance between the coil and the shield was 2 cm. For the ^2^H frequency, the ^2^H/^31^P whole‐body birdcage transmit coil was driven by a single‐channel, 10 kW RF amplifier (AN8112, Analogic Corporation, Peabody, MA, USA), using a quad hybrid to drive the birdcage in quadrature mode. For the ^31^P frequency, a two‐channel, 2 × 18 kW RF amplifier (AN8137, Analogic Corporation, Peabody, MA, USA) was connected to the ports of the birdcage.

Data were acquired on a 7 T whole‐body MR system (Philips Healthcare, Best, the Netherlands), using an dual tuned eight‐channel ^2^H/^31^P receive array with eight fractioned transmit/receive ^1^H dipole antennas [28] (WaveTronica, Utrecht, the Netherlands). The ^2^H/^31^P receive array consisted of eight separate elements. Within each element, a ^2^H/^31^P receive loop (265 mm in length, 155 mm in width) was combined with a fractioned ^1^H dipole antenna. ^2^H/^31^P receive loops were connected to an in‐house developed receiver box, and the loops were detuned during RF transmit. The ^1^H fractionated dipole antennas were connected to 8‐channel multitransmit system operating at 2 kW peak power per channel.

### 2.2 EM Simulation

**FIGURE 2:**
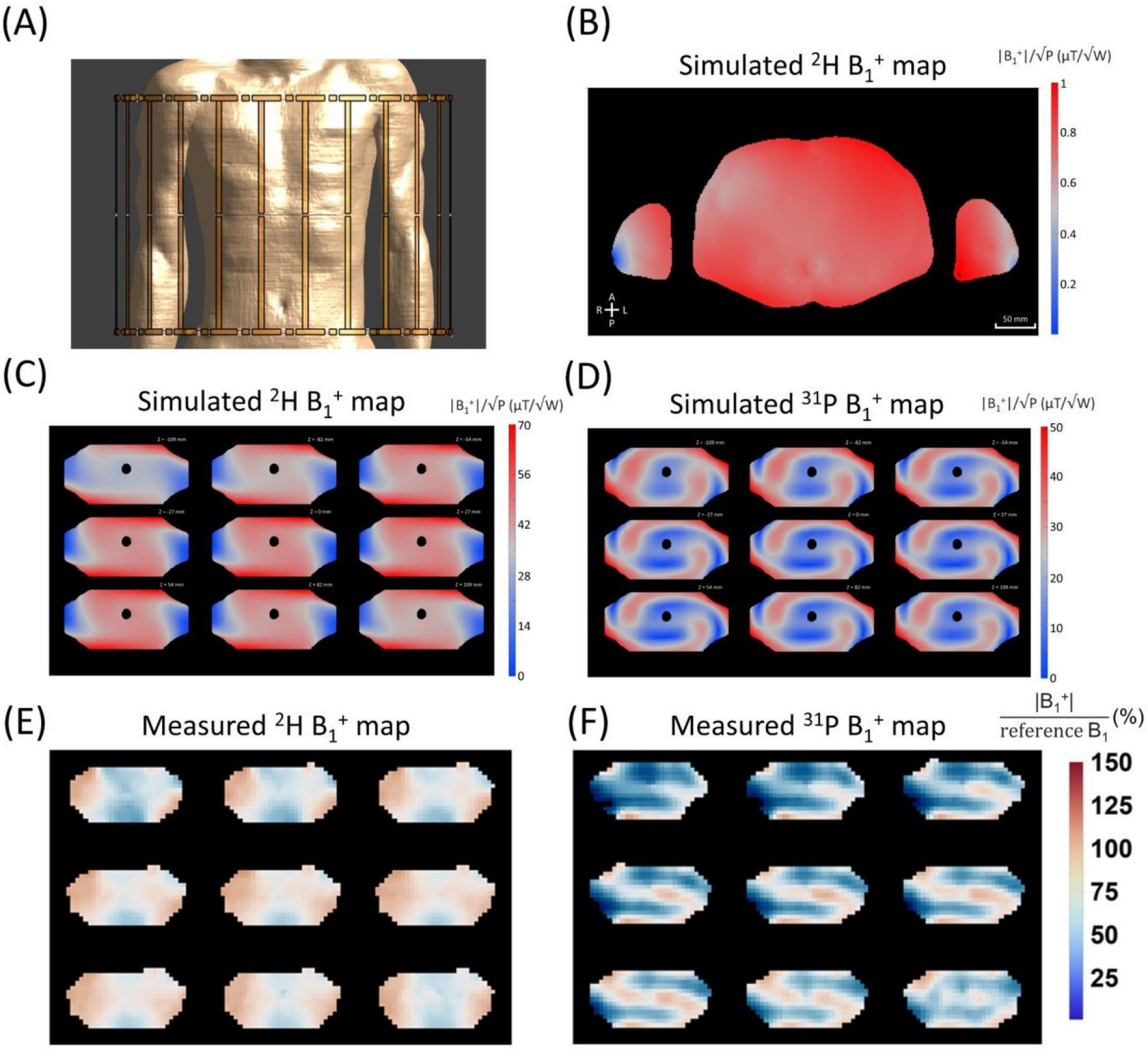
Results of electromagnetic (EM) simulations and phantom measurements with the double tuned ^2^H/^31^P birdcage coil. (A) Coil positioning on the Duke model in EM simulations shown in coronal view. (B) Simulated axial ^2^H B_1_^+^ map (|B_1_^+^|/√P) at the center of the liver. EM simulations on body phantom for ^2^H and ^31^P frequencies are shown in (C) and (D). ^2^H (E) and ^31^P (F) AFI B_1_^+^ maps of the double tuned ^2^H/^31^P whole‐body birdcage transmit coil measured on a body size phantom. Data are shown for 9 consecutive axial slices from the center of the body phantom. The reference B_1_^+^ was 10 μT for both ^2^H and ^31^P. The color bar indicates the percentage of this reference B_1_^+^.

In addition to extensive electromagnetic (EM) simulations available for 120.6 MHz in human models (i.e., close to ^1^H frequency of 3 T) [12, 29, 30], RF field homogeneity of the birdcage coil was evaluated with EM simulations (Sim4Life, ZMT, Zürich, Switzerland) at 45.7 MHz on the multitissue model Duke [31] (IT'IS Foundation, Zürich, Switzerland). Simulations were carried out at a resolution of 1.5 × 1.5 × 1.5 mm^3^ to render the birdcage coil and Duke model, resulting in 69.1 million mesh cells. The birdcage coil was placed such that the center of the birdcage coil was matched to the center of the liver (Figure 2A). In the simulations, the rungs and end rings were made of perfect electric conductive material, tuned to 45.7 MHz, matched to 50 Ω. Additionally, EM simulations were conducted on a body‐sized phantom (ε = 74, σ = 0.55 S/m), at frequencies of 45.7 and 120.6 MHz to compare the performance of double‐tuned birdcage coils in terms of homogeneity in both simulations and B_1_^+^ measurements (see next section). Material losses caused by double‐tuning LCC traps and rungs were not taken into account. The B_1_^+^ field was simulated in quadrature mode for 1 W total input power over the ports of the birdcage coil. The B_1_^+^ values in the abdomen were averaged over a slab in the center with a thickness of 20 cm, which covered the whole liver in the feet‐head axis. B_1_^+^ variation was reported as the percentage of the standard deviation of B_1_^+^ over the mean of B_1_^+^ (SD/mean). Additionally, the transmit efficiency of the birdcage coil was evaluated as mean B_1_^+^ of the abdomen over peak local SAR (mean(B_1_^+^)/√pSAR_10g_).

### 2.3 Phantom and B_1_^+^ Mapping

^2^H and ^31^P B_1_^+^ maps were acquired on a body size phantom (25.5 L) containing 3 g/L NaCl, 43 mM KH_2_PO_4_, and 0.54% D_2_O, using the actual flip angle imaging (AFI) method [32] (3D gradient echo:FOV = 360 × 480 × 360 mm^3^, resolution = 30 × 30 × 30 mm^3^, ^2^H/^31^P: TE = 2.5/1.03 ms, TR_1_ = 50/50 ms, TR_2_ = 650/950 ms, nominal flip angle = 65°/60°, reference B_1_^+^ = 10 μT, NSA = 12/64). The scanning parameters in the AFI sequence were optimized for the highest SNR per unit time by calculation of the Ernst angle. Considering long T_2_ relaxation times of inorganic phosphate [33] and in vitro deuterated water [15], spoiler gradients were set to maximum to ensure the dephasing of the remaining transverse signal. B_1_^+^ maps were reconstructed using QMRI tools [34].

### 2.4 In Vivo ^2^H and ^31^P MRSI Measurements at Natural Abundance

The study was approved by the local medical ethics committee and before the start of the acquisition, every participant gave their informed written consent. ^2^H and ^31^P 3D MRSI measurements of the brain and lower leg muscles were performed on one healthy volunteer (female, 34 years) at natural abundance and rest. For these two acquisitions, only two elements of the array were used. These elements were positioned at the posterior side of the brain and right lower leg. Using 2 ^1^H dipole antennas, a 3D B_0_ map (3D gradient echo, TE = 1.74 ms, ΔTE = 1.0 ms, TR = 10 ms, brain FOV (AP × RL × FH) = 240 × 240 × 141 mm^3^, beg FOV = 240 × 150 × 240 mm^3^) was acquired for image‐based B_0_ shimming. First‐ and second‐order shim settings were optimized over a volume of interest (VOI) using the MRCode software (TeslaDC, Zaltbommel, the Netherlands) [35]. For the brain scan, the VOI contained the posterior part of the brain (defined by threshold based brain extraction tool of the software). For the lower leg, the manually drawn VOI included skeletal muscles, while subcutaneous fat and tibia bone were avoided. Axial and coronal T_1_w anatomical reference images (2D multislice gradient echo) were acquired with the same FOV and the same number of slices as for the ^2^H and ^31^P MRSI scans. For both nuclei, a 3D MRSI sequence was used with a block excitation pulse and Hamming‐weighted k‐space acquisition (see Table 1 for acquisition parameters). FOV and nominal voxel size were identical for both nuclei. ^2^H and ^31^P MRSI were sequentially acquired without repositioning of subject.

**TABLE 1:** Acquisition parameters for in vivo ^2^H and ^31^P MRSI scans.

Tissue	Brain		Liver		Lower leg	
Nucleus	^2^H	^31^P	^2^H	^31^P	^2^H	^31^P
TR (ms)	100	60	100	60	100	60
TE (ms)	1.57	0.50	1.37	0.50	1.70	0.50
Nominal flip angle (°)	40	12	40	12	40	12
NSA	36	64	4	20	60	60
Total scan duration (min:sec)	15:04	15:23	13:25	20:59	23:44	14:13
Field of view LR×AP×FH (mm^3^)	200 × 220 × 300	200 × 220 × 300	500 × 300 × 340	500 × 300 × 340	154 × 140 × 350	154 × 140 × 350
Nominal voxel size LR×AP×FH (mm^3^)	20 × 20 × 20	20 × 20 × 20	20 × 20 × 20	20 × 20 × 20	14 × 14 × 25	14 × 14 × 25
Spectral bandwidth (Hz)	5000	5000	2750	5000	5000	5000
Spectral points	256	256	256	256	256	256

Abbreviations: NSA, number of averages; TE, echo time; TR, repetition time.

### 2.5 Liver ^2^H and ^31^P MRSI Measurements After [6,6′‐^2^H_2_]‐Glucose Intake

For liver ^2^H and ^31^P 3D MRSI measurements, one healthy volunteer (male, 29 years of age) was scanned after an overnight fast. [6,6′‐^2^H_2_]‐Glucose (20 g; Buchem B.V., Apeldoorn, the Netherlands) was dissolved in ~200 mL water and administered orally.

For this experiment, all eight elements of the receiver array were used and positioned around the abdomen; 4 at the anterior side and 4 at the posterior side, while keeping the arms of the subject next to the body. The center of the array elements was aligned to the center of the liver in the feet‐head direction.

For B_0_ shimming, a 3D B_0_ map (3D gradient echo, TE = 1.49 ms, ∆TE = 1 ms, TR = 10 ms, FOV = 280 × 502 × 147 mm^3^, acquisition time = 17.1 s) was acquired during a breath‐hold in the exhaled state. Shimming was performed across a VOI that included the entire liver. However, during optimization of the shim settings, the entire body was also taken into account of the cost function, albeit with less weighting [35]. Axial and coronal T_1_w anatomical reference images (2D multislice gradient echo) were acquired with the same field of view and the same number of slices as for the ^2^H and ^31^P MRSI scans (see Table 1 for acquisition parameters). FOV and nominal voxel size were identical for both nuclei. Acquisition of the ^2^H MRSI scan was started 90 min after glucose intake. ^31^P MRSI acquisition started immediately after the ^2^H MRSI scan.

### 2.6 Signal Processing and Analysis

MRSI data were reconstructed in MATLAB 2022a (The MathWorks Inc., Natick, MA).

The Fourier transformation was applied across the spatial domains, which was succeeded by zero‐order phase correction. PCA denoising was applied before Roemer equal noise channel combination [36, 37]. For visualization purposes, spectra were apodized with a 5‐Hz exponential function and zero‐filled to 2048 points.

## 3 Results

EM simulation results and measured B_1_^+^ maps of the ^2^H/^31^P whole‐body birdcage transmit coil are shown in Figure 2B showing that B_1_^+^ variation was 7.7% in Duke model. Transmit efficiency of the birdcage coil, reported as mean(B_1_^+^)/√pSAR_10g_, was 1.59. Simulated B_1_^+^ maps in the body phantom are shown (Figure 2C,D). In the EM simulations, B_1_^+^ variations over the body phantom were 13.3% and 37.6% for ^2^H and ^31^P, respectively. Measured B_1_^+^ over the whole phantom was 9.64 ± 1.16 μT (Figure 2E) and 8.32 ± 1.98 μT (Figure 2F) for ^2^H and ^31^P frequencies, respectively. When scaled to the RF input power, measured B_1_^+^ for ^2^H and ^31^P became 0.163 ± 0.016 μT/√W and 0.055 ± 0.013 μT/√W, respectively. The B_1_^+^ variation within the phantom was 12.1% for ^2^H and 19.2% for ^31^P.

**FIGURE 3:**
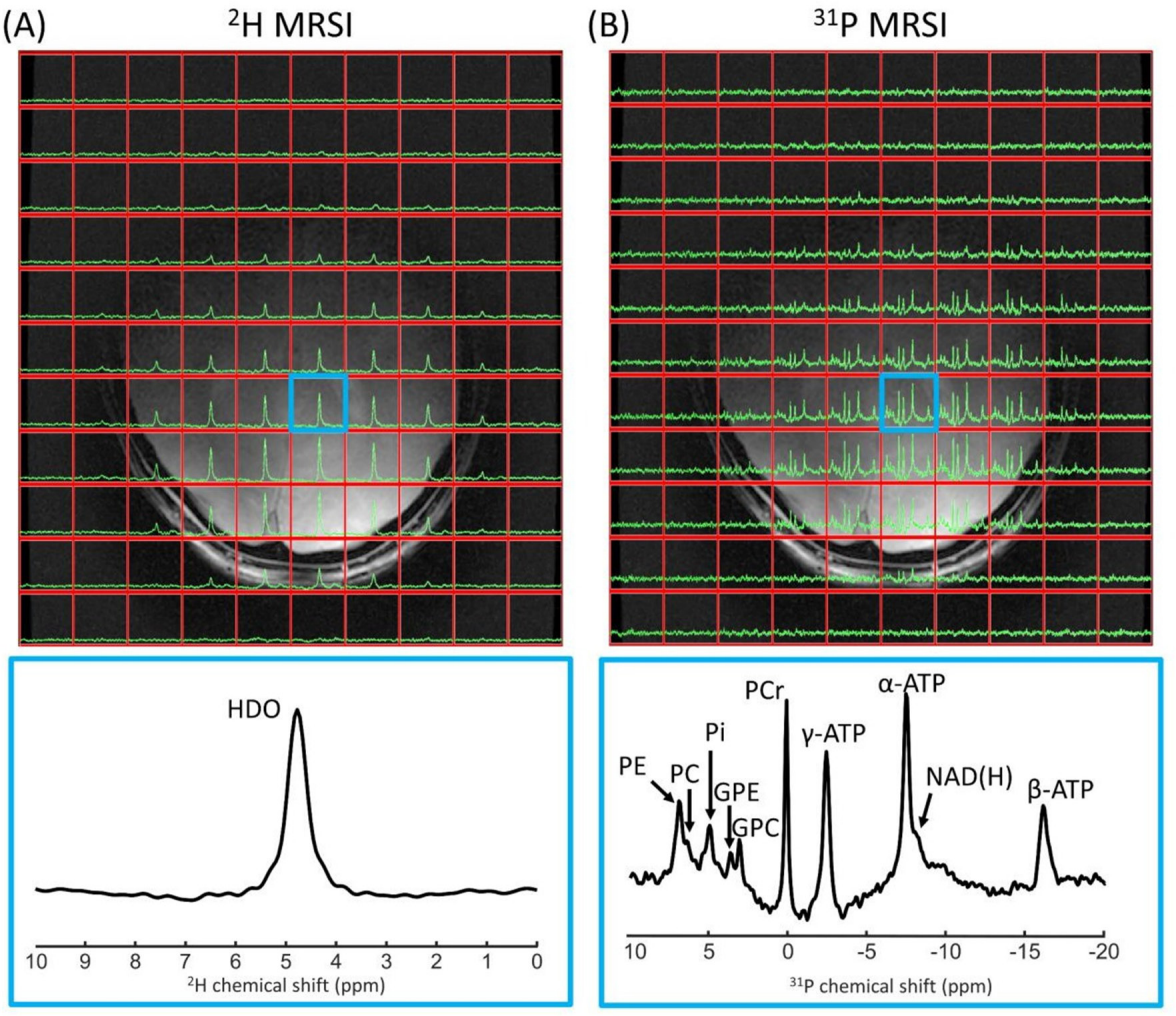
One slice of natural abundance ^2^H 3D MRSI (A) and ^31^P 3D MRSI (B) data of the brain overlaid on the T1w image, together with enlarged spectra from a selected voxel (indicated in blue). ^2^H/^31^P spectra were apodized with 20 Hz and zero‐filled to 2048/512 points for visualization purposes. GPC, glycerophosphocholine; GPE, glycerophosphoethanolamine; HDO, deuterated water; NAD(H), nicotinamide adenine dinucleotide; PC, phosphocholine; PCr, phosphocreatine; PE, phosphoethanolamine; Pi, inorganic phosphate.

**FIGURE 4:**
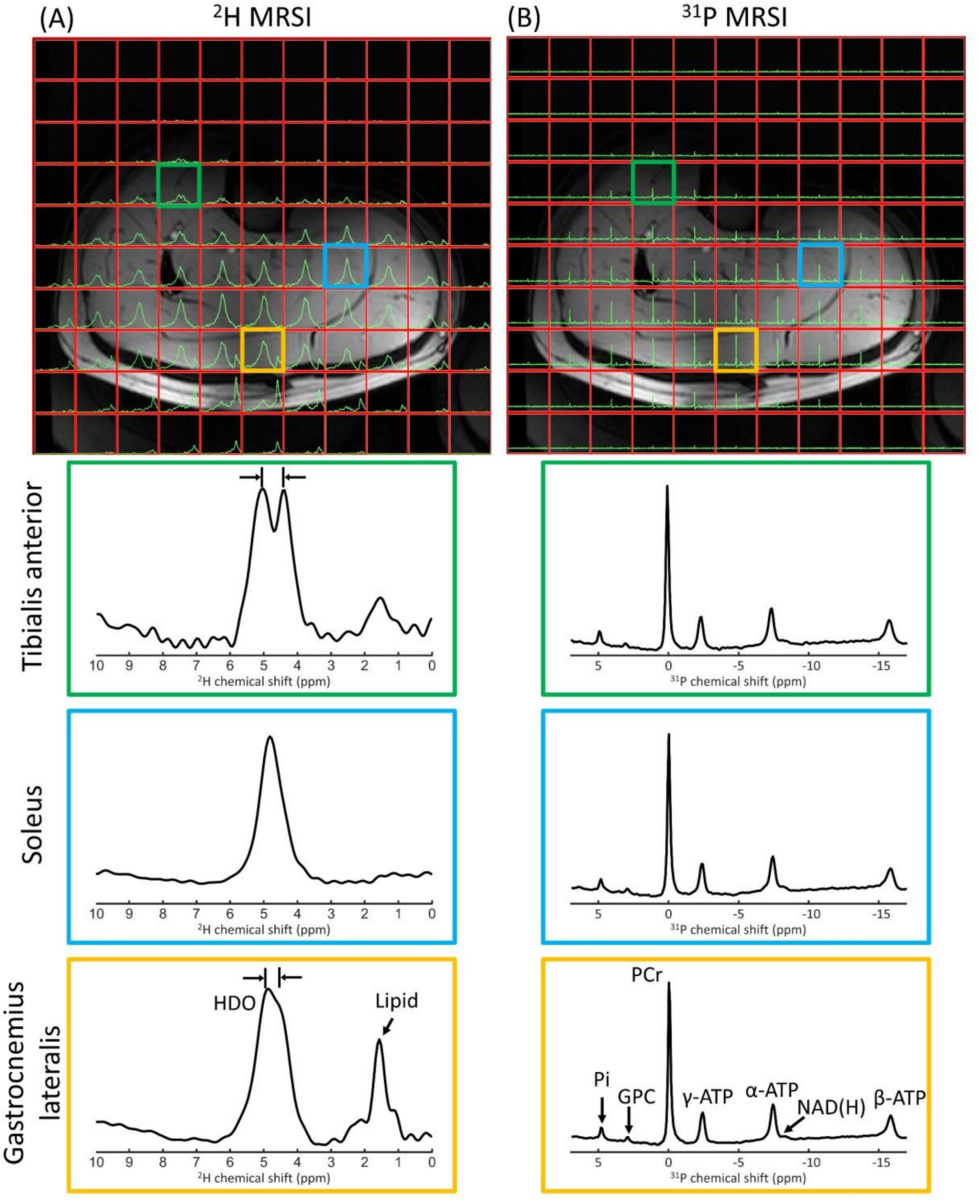
One slice of natural abundance ^2^H 3D MRSI (A) and ^31^P 3D MRSI (B) data of the lower leg overlaid on the T1w image, together with enlarged spectra from selected voxels in the tibialis anterior, soleus and gastrocnemius lateralis (green, blue, and orange) muscles. ^31^P spectra were apodized with 20 Hz, and ^2^H/^31^P spectra were zero‐filled to 2048/512 points for visualization purposes. The enlarged spectra for the different voxels were rescaled to equal signal intensity. In the tibialis and gastrocnemius ^2^H spectra, the deuterated water signal is split due to residual quadrupolar couplings [38].

In vivo ^2^H and ^31^P 3D MRSI data of the brain and lower leg are shown in Figure 3 and Figure 4. The ^2^H spectra of the brain showed a signal from the natural abundance deuterated water, with additional signals from lipids for voxels close to the skull. The muscle deuterated water signals showed residual quadrupolar couplings in tibialis anterior and gastrocnemius lateralis muscles. While high‐energy phosphates were detected in both brain and lower leg ^31^P spectra, phosphomonoesters (PME) and/or phosphodiesters (PDE) were detected more clearly in brain ^31^P spectra.

**FIGURE 5:**
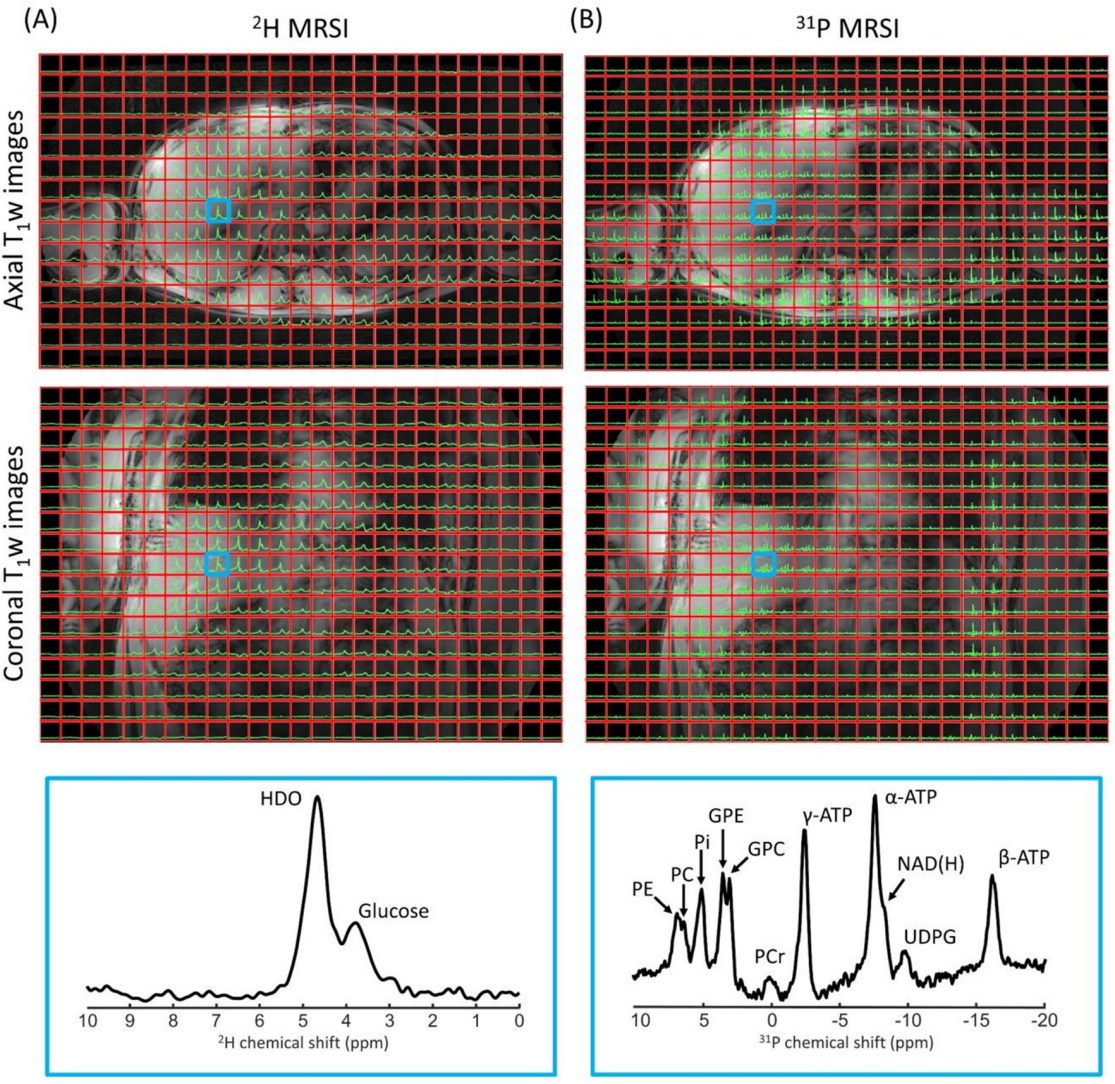
One slice of ^2^H 3D MRSI (A) and ^31^P 3D MRSI (B) data of the liver overlaid on the axial and coronal T_1_w images, together with enlarged spectra from a selected voxel (indicated in blue). ^2^H 3D MRSI data were acquired 90 min after oral administration of [6,6′‐^2^H_2_]‐glucose and the ^31^P 3D MRSI were acquired directly thereafter, without repositioning of the subject. ^31^P spectra were apodized with 20 Hz and spectra from muscle were clipped along the vertical axis in order the visualize the smaller signals in the liver. ^2^H/^31^P spectra were zero‐filled to 2048/512 points. PtdC, phosphatidylcholine; UDPG, uridine diphosphate glucose.

Liver ^2^H and ^31^P 3D MRSI data are shown in Figure 5. Liver ^2^H MRSI data were acquired 90 min after oral intake of 20 g of [6,6′‐^2^H_2_]‐glucose, and besides, the deuterated water signal, a signal from deuterated glucose signal was detected at 3.8 ppm within the whole liver. In the liver ^31^P spectra, high‐energy phosphates were detected together with uridine diphosphate glucose (UDPG), PME, and PDE.

## 4 Discussion and Conclusion

In this study, we developed a double tuned whole‐body birdcage transmit coil for ^2^H and ^31^P at 7 T. B_1_^+^ maps for ^2^H and ^31^P were acquired using a body size phantom and characteristics of the B_1_^+^ fields were assessed in terms of field homogeneity. We demonstrated the feasibility of obtaining ^2^H and ^31^P 3D MRSI data throughout the body with this setup, with examples in the brain, liver, and lower leg.

For the ^2^H frequency, the B_1_^+^ field of the birdcage coil was very homogeneous in both the simulations (7.7% and 13.3% variation for Duke and body phantom, respectively) and the phantom measurement (12.1% variation), as expected for this frequency (45.7 MHz, i.e., similar to ^1^H frequency at 1 T). For ^31^P (i.e., 120.6 MHz), the B_1_^+^ field was more inhomogeneous (37.6% and 19.2% variation in simulation and measurement), but comparable with what has been simulated and observed for ^1^H whole‐body coils at 3 T (128 MHz) [29, 30, 39, 40]. The measured ^31^P B_1_^+^ maps from the body phantom resemble the simulated B_1_^+^ field of a ^1^H body coil at 128 MHz, in an axial slice in the abdomen, seen in the literature [29, 30]. However, the observed nonuniformity patterns in the measured ^31^P B_1_^+^ maps are more pronounced compared with the simulated ^1^H B_1_^+^ maps, which is likely due to the high conductivity (0.55 S/m) and high relative permittivity (78) of the phantom fluid. Additionally, for both ^2^H and ^31^P frequencies, the B_1_^+^ variation in the body phantom simulations was higher than in the measurements, primarily due to the significantly higher resolution in the electromagnetic simulations. In the ^2^H measurements, the B_1_^+^ magnitude of the double‐tuned birdcage coil dropped by only 20% at the center of the body phantom (Figure 2E), compared with a 50% drop observed with a four‐channel ^2^H transmit/receive body array coil [41]. For the ^31^P frequency B_1_^+^ simulations, the double‐tuned birdcage coil performed similarly compared with preliminary results of an 8‐channel array consisting of double tuned (^31^P/^1^H) dipoles, on a body phantom [42].

When scaled to input RF power, B_1_^+^ amplitude for ^2^H frequency became 0.163 ± 0.016 μT/√W. This value is lower than the 0.227 μT/√W reported for a 1.5 T RF body coil using the same phantom [43]. The lower B_1_^+^ amplitude in our setup may be attributed to RF power losses in the LCC circuits in the end rings [27]. For simplicity in design and avoid further power losses, transmit detuning circuitry was not implemented. In combination with an array of receivers that are equipped with preamplifier decoupling, the noise coupling will be low [44]. Moreover, as the body coil is compromised in efficiency due to double tuning, the noise coupling will be further reduced. Noise correlation measurements between all receiver elements inside versus outside the body coil indeed did not show a significant difference. There may be situations (i.e., large receiver loops close to the end rings of the body coil) where noise coupling becomes nonnegligible. In these cases, either active transmit detuning should be considered or transmit‐receive switches to also obtain the signal from the body coil allowing noise decorrelation in the image reconstruction [44].

For the brain and lower leg acquisitions, where we used only two receive loops at the posterior side, signal intensity decreased at larger distances to the receive elements (from posterior to anterior), but for most of the brain and lower leg, clear signals could be detected for both ^2^H and ^31^P. Because ^2^H acquisitions were made at natural abundance, only deuterated water and, for voxels located near adipose fat tissue, lipid peaks were detected. Additionally, in lower leg ^2^H spectra, residual quadrupolar couplings were detected in voxels located within the tibialis anterior and gastrocnemius lateralis muscles where muscle fibers run in parallel to the B_0_ field [38]. In contrast, muscle fibers in the soleus muscle are angled to the B_0_ field axis, and splitting of the deuterated water peak was not observed.

The combination of ^2^H and ^31^P MRSI in the liver after oral intake of [6,6′‐^2^H_2_]‐glucose could provide unique insight into hepatic glucose and energy metabolism in the postprandial state in a single scan session. In contrast to previous liver ^2^H MRSI studies [1, 41, 45], we were able to achieve full liver coverage. Moreover, our scans could be obtained at short TR and Ernst angle excitation without the need for adiabatic RF pulses ensuring optimized SNR per unit of time without being hindered by SAR limits [46]. Although ^2^H‐labeled glycogen is MR invisible because of its short T_2_ relaxation time [47], UDPG, detectable by ^31^P MRSI, could potentially be used as a biomarker of hepatic glycogenesis [48]. In a similar effort to our study, Poli et al. combined ^2^H and ^13^C MRS in interleaved acquisitions to monitor dynamic glucose uptake and glycogen levels in the liver [45]. Recently, van den Wildenberg et al. showed metabolic alterations in liver metastases and the impact of chemotherapy on cancer tissue by using 3D ^31^P MRSI [49]. Addition of ^2^H MRSI with [6,6′‐^2^H_2_]‐glucose to this application could potentially improve monitoring capabilities during chemotherapy by introducing a complementary biomarker, deuterated lactate, to PDE and PME, which are biomarkers for cell proliferation.

Finally, as limited penetration depth of local Tx coils is not an issue for the developed setup, monitoring metabolic activity within the whole liver or deep tissues in the abdomen, such as the pancreas, becomes possible with ^2^H and ^31^P MRSI. As an example, this could allow a deeper understanding of pancreatic tumors that were previously studied with ^2^H MRSI [50] in a preclinical setup and with ^31^P MRSI [26] in a patient with pancreatic ductal adenocarcinoma.

In the current study, a ^31^P/^2^H receiver array was designed for abdominal acquisitions and using only two elements of the array hindered spatial coverage when used on the leg and brain. This was especially true for the frontal part of the brain. The dimensions of the receive elements were optimized for the body. As placing the receive elements too close to each other may result in high coupling between the elements, smaller parts of the body, such as the head and the lower leg, were scanned with only two receive elements. Dedicated receive setups can be developed for specific applications, to be used in conjunction with the whole‐body birdcage transmit coil, as is common for ^1^H applications at clinical field strengths. To enhance B_1_^+^ efficiency, new double tuned birdcage coil designs with a reduced number of LCC circuits may be developed to reduce loss in LCC circuits [51]. Furthermore, the installation of high power RF switches or combiners between RF amplifiers and the birdcage coil would allow rapid interleaved ^2^H and ^31^P acquisitions, which could shorten the total acquisition time [52].

In conclusion, we developed a double tuned ^2^H/^31^P whole‐body birdcage transmit coil for 7 T that allows the combined application of ^2^H and ^31^P MRSI throughout the human body, for simultaneous 3D mapping of glucose energy metabolism and membrane turnover.

## Data Availability

The data that support the findings of this study are available from the corresponding author upon reasonable request.
